# Alexander Duncan Langmuir

**DOI:** 10.3201/eid2109.141445

**Published:** 2015-09

**Authors:** Myron G. Schultz, William Schaffner

**Affiliations:** Centers for Disease Control and Prevention, Atlanta, Georgia, USA (M.G. Schultz);; Vanderbilt University School of Medicine, Nashville, Tennessee, USA (W. Schaffner)

**Keywords:** Epidemic Intelligence Service, disease surveillance

**Figure 1:**
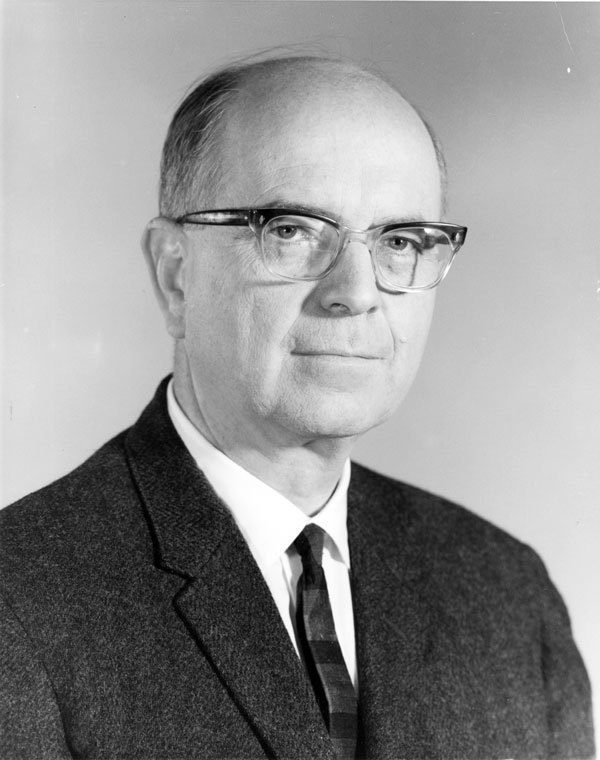
This is a photograph of Alexander Duncan Langmuir (September 12, 1910–November 22, 1993). Langmuir, a renowned epidemiologist who created the Epidemic Intelligence Service (EIS), developed the practice of modern public health surveillance in the United States and abroad.

Alex Langmuir was born in Santa Monica, California, and grew up in New Jersey. His uncle, Irving Langmuir, a physicist and chemist, won the Nobel Prize in Chemistry in 1932. At Harvard College, Alex Langmuir tried to follow in his uncle’s footsteps, but he found that the mathematics of advanced physics was beyond him and thus decided to pursue a career in medicine. He received his AB (cum laude) in 1931 from Harvard and his MD in 1935 from Cornell University Medical College. As a college student, Langmuir was inspired by Massachusetts Commissioner of Health George Hoyt Bigelow to enter the field of public health. His first 2 jobs were with the New York State Health Department; he began as a medical consultant and then became an assistant district health officer in Albany. After graduating with an MPH from the Johns Hopkins School of Hygiene and Public Health in 1940, Langmuir became a deputy commissioner of health in Westchester County, New York. His family was dismayed that he chose a career in public health rather than clinical medicine, but Langmuir expressed in his later years that his time in local public health taught him lessons that were fundamental to his achievements. From 1942 to 1946, he served as an epidemiologist with the Armed Forces Epidemiologic Board’s Commission on Acute Respiratory Diseases, stimulating his lifelong interest in influenza. In 1946, Langmuir returned to Johns Hopkins University as an associate professor of epidemiology. However, by 1949 he was restive in academia and was attracted to the challenge of becoming the first chief epidemiologist of the newly established Communicable Disease Center (now the Centers for Disease Control and Prevention [CDC]) in Atlanta, Georgia, a position he held for over 20 years. When Langmuir retired from CDC, he became a visiting professor of epidemiology at Harvard Medical School and, later, a visiting professor of epidemiology at Johns Hopkins School of Hygiene and Public Health. He wrote extensively on all phases of epidemiology and public health surveillance on a global basis and was recognized internationally as an assertive public health authority.

In 1951, following the start of the Korean War, Langmuir established the EIS program as an early warning system against biologic warfare. EIS officers then and now are physicians, veterinarians, nurses, and health scientists who serve 2-year assignments. In an obituary written for the *New York Times*, Lawrence Altman said Langmuir “taught what he called ‘shoe leather epidemiology,’ stressing that investigators go into the field to collect their own data and view directly the locale of the public health problem they were investigating.” Langmuir said: “Each epidemic aid call was an adventure and a training experience, even the false alarms.” He stressed that field epidemiology should be taught in the field, not in the classroom. Admission into the EIS program was highly selective. Langmuir believed that when competent persons were thrust into challenging circumstances with supportive supervision, excellent results were certain. He regarded the EIS officers as members of his extended family, backing them firmly when they found themselves in difficulty and joining them for the roasts of CDC leaders during the officers’ annual skit night—often at his own expense.

In 1955, Langmuir and his young staff achieved early recognition due to the “Cutter Incident.” The new inactivated (Salk) polio vaccine was causing cases of polio. Surgeon General Leonard Scheele asked Langmuir to develop a surveillance system to determine the extent of the problem. Langmuir deployed his staff, and within days they determined that the cases were caused by vaccine from a single manufacturer: Cutter Laboratories. “Langmuir was able to predict with great accuracy the expected size of the epidemic and the number of secondary cases that would occur,” former CDC director William Foege noted. This response enhanced the reputation of the young agency and established epidemic aid as one of its singular characteristics.

Today, the EIS program has evolved into a surveillance and response unit for all types of health problems. During 1951–2014, more than 3,500 physicians, veterinarians, nurses, and health scientists were trained as EIS officers. Many of the nation’s medical and public health leaders, including CDC directors, state health department directors, state epidemiologists, and deans of the country’s premier schools of public health, are EIS alumni. Since 1980, CDC has supported the development and implementation of 48 two-year field epidemiology training programs that cover 60 countries and are modeled after the EIS in their teaching and practice of applied field epidemiology. More than 3,000 epidemiologists have graduated from these programs; many of these graduates now hold leadership positions within their countries’ ministries of health, the World Health Organization, and other global health organizations.

The idea of effective national disease surveillance captured Alex Langmuir’s imagination throughout his career. He believed that surveillance is the foundation for evidence-based public health action. Langmuir preached the importance of the systematic collection of pertinent data, the consolidation and analysis of these data into useful information, and the dissemination of the results to all who need to know so that they can take action. His goal was to use surveillance systems to define populations at risk for disease, determine interventions, and monitor their impact. Langmuir and his staff developed novel national surveillance programs for an array of communicable diseases and for chronic diseases, injuries, and reproductive health. Indeed, he considered the population explosion to be the most serious epidemic of all.

Altman described Langmuir as “a tall man who could command immediate attention when he stood to speak to audiences in his deep voice. He thrived on controversy and took pride in overcoming local political pressures to crusade for preventive medicine and other measures to safeguard public health.” Philip Brachman, who succeeded Langmuir as EIS director, described Langmuir as “visionary, clairvoyant, tenacious, well prepared, scientifically honest, and optimistic.” Langmuir enjoyed being a civil servant and working to benefit the public. “His concerns were to control and prevent disease by applying the principles of epidemiology to the identification of causes and solutions,” Brachman wrote. Foege described Langmuir as someone with a public health message who arrived at the right time and place in history to be able to broadly disseminate his message.

In 1979, when Alex Langmuir was interviewed by D.A. Henderson about being recruited to work at CDC in 1949, Langmuir said, “As I looked it over and saw the vision, there was no question, [former CDC director] Justin Andrews took me to the mountain and showed me the Promised Land.” At CDC, Alex Langmuir changed the way epidemiology is used in public health practice, first in the United States and then throughout the world. In the 65 years since Langmuir’s arrival at CDC, his disciples—EIS and field epidemiology training program officers—have played pivotal roles in combating the root causes of major public health problems. Millions of persons live longer and healthier lives because of the accomplishments of Langmuir and his progeny in controlling and preventing disease. This is Alex Langmuir’s grand legacy.

## References

[1] A tribute to Alexander D. Langmuir. Am J Epidemiol. 1996;144(8 Suppl):1 p preceding S1, S1–78. 8928703

[2] Altman LK. Alexander Langmuir dies at 83; helped start U.S. disease centers. New York Times [cited 2015 June 23]. http://www.nytimes.com/1993/11/24/obituaries/alexander-langmuir-dies-at-83-helped-start-us-disease-centers.html

[3] Brachman PS. Epilogue: Alexander Duncan Langmuir. Am J Epidemiol. 1996;144(8 Suppl):S74–5. 10.1093/aje/144.Supplement_8.S74 8857846

[4] Foege WH, Alexander D. Langmuir—his impact on public health. Am J Epidemiol. 1996;144(8 Suppl):S11–5. 10.1093/aje/144.Supplement_8.S11 8857836

[5] Langmuir AD. The surveillance of communicable diseases of national importance. N Engl J Med. 1963;268:182–92. 10.1056/NEJM196301242680405 13928666

[6] Langmuir AD. The Epidemic Intelligence Service of the Center for Disease Control. Public Health Rep. 1980;95:470–7. 6106957 PMC1422746

[7] Langmuir AD, Henderson DA. Leaders in American medicine. Videotape series. Menlo Park (CA): Alpha Omega Alpha Honor Medical Society; 1979.

